# Auditory–Perceptual and Auditory–Motor Timing Abilities in Children with Developmental Coordination Disorder: A Scoping Review

**DOI:** 10.3390/brainsci13050729

**Published:** 2023-04-27

**Authors:** Marija Pranjić, Niloufaralsadat Hashemi, Anne B. Arnett, Michael H. Thaut

**Affiliations:** 1 Music and Health Science Research Collaboratory, University of Toronto, Toronto, ON M5S 1C5, Canada; 2 Bloorview Research Institute, Holland Bloorview Kids Rehabilitation Hospital, Toronto, ON M4G 1R8, Canada; 3 Institute of Biomedical Engineering, University of Toronto, Toronto, ON M5S 3G9, Canada; 4 Division of Developmental Medicine, Boston Children’s Hospital, Boston, MA 02115, USA; 5 Pediatrics, Harvard Medical School, Boston, MA 02115, USA; 6 Faculty of Medicine, Institute of Medical Science and Rehabilitation Research Institute, University of Toronto, Toronto, ON M5S 1A8, Canada

**Keywords:** developmental coordination disorder (DCD), dyspraxia, neurodevelopmental disorders, auditory–motor timing, motor difficulties, time perception

## Abstract

Developmental coordination disorder (DCD) remains largely underdiagnosed and masked by other co-occurring conditions. The aim of this study was to (1) provide the first review of research regarding auditory–motor timing and synchronization abilities in children with DCD and (2) examine whether reduced motor performance may be associated with difficulties in auditory perceptual timing. The scoping review was carried out across five major databases (MEDLINE, Embase, PsycINFO, CINAHL, and Scopus) in accordance with the PRISMA-ScR guidelines. Studies were screened by two independent reviewers against the inclusion criteria, without publication date restrictions. From an initial return of 1673 records, 16 articles were included in the final review and synthesized based on the timing modality studied (i.e., auditory–perceptual, motor, or auditory–motor). Results suggest that children with DCD have difficulties with rhythmic movements both with and without external auditory cues and further indicate that variability in and slowness of motor response are key characteristics of DCD, regardless of the experimental task. Importantly, our review highlights a significant gap in the literature regarding auditory perceptual abilities in DCD. In addition to testing auditory perception, future studies should compare the performance of children with DCD on paced and unpaced tasks to determine whether auditory stimuli contribute to a more or less stable performance. This knowledge may inform future therapeutic interventions.

## 1. Introduction

Developmental coordination disorder (DCD) is one of the most common neurodevelopmental disorders, occurring in approximately 6% of school-age children [1,2]. DCD is characterized by marked difficulties in motor planning, fine and/or gross motor coordination, sensorimotor timing, as well as acquisition and transfer of new motor skills. The diagnosis of DCD is given if these difficulties interfere with activities of daily living and/or academic performance, while ensuring those differences cannot be better explained by other medical and/or neurological conditions [1]. Motor coordination difficulties in children significantly affect quality of life and lead to anxiety, lower self-esteem, social isolation, and reduced fitness levels [3,4,5]. Despite its high incidence, DCD has been described as a “hidden problem” [6] as it remains less studied and under-recognized by healthcare and educational professionals [7] compared to other neurodevelopmental disorders (NDDs).

NDDs impact approximately 15% of children worldwide, encompass a broad range of conditions that typically manifest early in life, and affect performance in different domains of functioning, including attentional, sensory, motor, linguistic, and social areas [1]. Difficulties in any of these domains rarely occur in isolation, thus the overlapping symptoms are the rule rather than the exception [8]. In addition to primary challenges associated with motor control, children with DCD demonstrate a range of NDD symptoms [8]. The phenotypic complexity of DCD poses additional challenges to understanding neurobiological mechanisms for DCD. Neuroimaging studies of DCD have largely focused on the cerebellum, due to its role in the development of automatic movement control and movement self-monitoring [9], both of which are affected in DCD [10,11]; however, emerging neuroimaging data provide evidence of atypical neural activations across both cortical and subcortical regions, including the prefrontal and parietal cortices, cerebellum, basal ganglia, and white matter alterations in the corpus callosum [12,13,14,15]. Although these studies have provided important insights into the neurophysiological substrates associated with motor difficulties, heterogeneity in DCD creates significant barriers to interpreting and translating those findings into individualized treatments. 

Several hypotheses have been proposed to explain the motor performance differences associated with DCD. The most prominent is the internal modelling deficit hypothesis [16,17] which suggests that the difficulties in motor learning observed in children with DCD result from a deficiency in generating or implementing predictive models of action (forward modelling), such as correcting actions in real-time [18,19]. In other words, predictive timing allows for error detection and correction of the ongoing movement in situations when actual and predicted feedback do not correspond. Considering sensory error feedback plays an important role in the development of internal models [20], it is not surprising that difficulties in sensorimotor timing are one of the core features of DCD. Indeed, previous studies have shown that children with DCD are less accurate and more variable when synchronizing their movement to an external cue compared to typically developing controls regardless of the modality of the stimuli [21,22,23]; however, given that integration of sensory input is a prerequisite for synchronized sensorimotor performance, the observed challenges may also arise from perceptual or perceptual-motor integration challenges. 

Thus, a new auditory–perceptual timing hypothesis has been postulated by Trainor et al. [24] who suggest that deficits in auditory perceptual timing might also be central to DCD. This hypothesis draws from research showing that even passive listening to auditory stimuli with predictive rhythmic beats leads to activations in motor-related brain areas [25,26,27]. Notably, this line of research has led to novel auditory-based therapeutic interventions in adults with movement disorders [28,29,30] that have shown that rhythmic auditory cues can facilitate planning and execution of movements by increasing temporal stability and speed. Furthermore, auditory perceptual timing abilities are affected in neurodevelopmental disorders associated with DCD, including attention-deficit/hyperactivity disorder, dyslexia, and autism spectrum disorder [31,32,33,34]. Therefore, a shared deficit in perceptual timing may explain the overlapping profile of motor difficulties across these relatively distinct neurodevelopmental phenotypes [35].

### Aims

The aim of this scoping review was to systematically map and synthesize the existing empirical research regarding auditory–motor coupling in children with DCD by focusing on three timing modalities that underpin this process: auditory–perceptual timing (perceptual judgment without movement), motor timing (self-paced actions), and auditory–motor timing (adjusting the timing of motor responses based on the auditory cue) [24]. We examined whether the variability of auditory–motor coupling shown by children with DCD is task-specific and whether their performance differences are associated with difficulties in auditory–perceptual timing.

## 2. Materials and Methods

The scoping review was conducted in accordance with the Joanna Briggs Institute methodological framework [36], using the Preferred Reporting Items for Systematic Reviews and Meta-Analyses Extension for Scoping Reviews (PRISMA-ScR) criteria: Checklist and Explanation guidelines [37]. We addressed the following questions: (1) What is known from the literature about the auditory–motor timing and synchronization abilities in children with DCD? (2) Do children with DCD have both auditory–perceptual and auditory–motor difficulties? The protocol was registered with the Open Science Framework on 3 March 2023 (https://doi.org/10.17605/OSF.IO/5KAXQ (accessed on 3 March 2023)).

### 2.1. Search Strategy

The population, concept, and context (PCC) mnemonic was used to formulate the search strategy. We used terms such as “DCD”, “motor skill disorder” or “dyspraxia” to define the population (i.e., individuals with DCD) combined with terms for the concept (i.e., auditory–motor timing) such as “auditory-motor coupling” or “synchronization”. The context was not specified, in order to capture all relevant publications regardless of setting (e.g., clinic or research laboratory). Subject headings were adapted for each database. The search strategy was developed and performed by two authors (M.P. and N.H.) with assistance from a research librarian. A scoping review was carried out across five major databases (MEDLINE, Embase, PsycINFO, CINAHL, and Scopus) for articles published before April 2022. Additionally, we cross-referenced the bibliographies of selected studies to ensure we captured current and emerging evidence. Table 1 provides an example of the search strategy performed in MEDLINE (see Supplementary Table S1 for the full electronic search strategy). The appropriate filtering processes of the final search results were done via Covidence.

### 2.2. Eligibility Criteria

Inclusion criteria were studies that (1) involved children (<18 years) with motor coordination difficulties (i.e., DCD or probable DCD as assessed with a standardized motor test), (2) examined the effects of auditory stimuli on motor performance, (3) were published in English, and (4) were categorized as original research. Since this is the first review on the topic, publication year was not considered as an inclusion/exclusion criterion; however, we recognize that the term “developmental coordination disorder” was introduced in 1994, resulting in a wide variation in terminology used prior to that year. Articles were excluded if the outcome measures were not related to motor performance and if the primary goal was an evaluation of a treatment method. All titles, abstracts, and full-text publications were screened by two independent reviewers for inclusion and exclusion using the relevance screening tool (see Supplementary Tables S2 and S3). Relevant studies were further evaluated and discrepancies between the reviewers regarding study selection and data extraction were resolved by consensus (<5 studies).

### 2.3. Data Extraction and Synthesis

We systematically extracted the following data: publication details, sample characteristics (i.e., age, sex, sample size, motor ability), study design (i.e., experimental tasks, timing modality), and outcomes. One reviewer (M.P.) extracted data from the included papers, refining and updating the data-charting form in the process. The second reviewer (N.H.) verified the correctness of the extracted information. Data from selected studies were collated, summarized, and reported based on the type of task (i.e., timing modality) used: (1) auditory–perceptual (perceptual judgment is assessed without producing movements), (2) motor (self-paced and continuation tasks), and (3) auditory–motor (motor responses are synchronized or adjusted based on the auditory cue). We aimed to organize the studies in this framework to establish commonalities between the types of tasks used, their underlying timing modalities, and the reported outcomes. The results were further discussed in the context of two dominant perspectives on motor timing: information processing and the dynamical systems theory. Finally, our findings are considered in the context of the auditory–perceptual timing hypothesis [24].

## 3. Results

**Figure 1:**
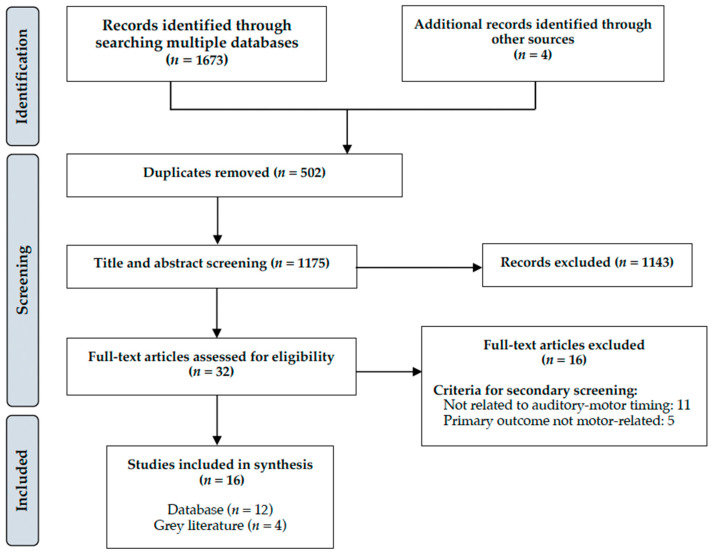
Selection of sources of evidence (PRISMA-ScR Flowchart).

As illustrated in the PRISMA-ScR flowchart in Figure 1, a total of 1673 sources were identified from searches of five electronic databases. Four additional studies were identified through manual search of the bibliographies. Based on the title and the abstract, 1143 articles were excluded and a total of 32 sources remained for full-text screening. Of these, 16 were excluded for the following reasons: 11 did not directly examine the effects of auditory stimuli on motor performance, and 5 did not report motor-related outcomes. Thus, a total of 16 studies were considered eligible for this review and are summarized in Table 2. All selected studies employed a cross-sectional design whereby participants performed experimental tasks at a single time point. The following sections synthesize results across the 16 studies and present findings organized by the timing modality studied and the experimental task used (note that some experiments involved more than one type of task or timing modality and are therefore discussed in more than one section).

### 3.1. Auditory–Perceptual Timing

Only three studies tested the auditory–perceptual abilities of children with DCD separate from their motor or auditory–motor performance [38,39,40]. Two studies used the same time perception task wherein children aged 7–8 years [38] and 6–7 and 9–10 years [39] listened to two pairs of tones and were asked to judge whether the interval of the second pair was longer or shorter than that of the first pair. The interval of the second pair changed adaptively based on the child’s response in order to hone in on the individual child’s perceptual threshold. A similar task, in which the children were expected to determine differences in volume between two pairs of tones was also administered to control for general auditory processing differences. Both studies found that the threshold scores for duration-based timing were longer (i.e., higher) in children described as “clumsy” compared to healthy controls, suggesting temporal processing difficulties in the former group. In contrast, volume thresholds did not differ between groups, indicating intact hearing. Williams et al. [39] further found that there were age differences for the loudness perception task wherein younger children (6–7 years) had lower acuity than the 9–10-year-old group regardless of motor performance. 

Interestingly, Lundy-Ekman et al. [38] compared two groups of “clumsy” children and reported that children with cerebellar soft signs (e.g., dysmetria, dysdiadochokinesia and/or intention tremor) exhibited increased difficulty with the duration timing task compared to the “clumsy” children with basal ganglia signs (e.g., synkinesis, choreiform movements and/or athetoid cerebral palsy) suggesting that the cerebellum may be the source of both perceptual and motor timing difficulties. However, these findings should be interpreted cautiously because the “clumsy” children described in publications before 1994 [7] may not meet the current DSM-5 or ICD-10 criteria for DCD [1].

In a more recent study, Roche et al. [40] found no significant differences in the perceptual thresholds for beat-based timing between 6–11-year-old children with and without DCD; however, it is important to note that the threshold estimation procedure used in this study was incongruous with that which is commonly used in adaptive psychophysical measurements [41]. First, Roche et al. [40] used a simple up-down staircase procedure where one positive or negative response influenced the level of difficulty on the subsequent trial. Compared to the more robust two- or three-up procedures [42,43], the one-up algorithm targets only the 50% level of performance. Second, the most common way of estimating perceptual thresholds for the staircase procedure is by averaging the responses or turnaround points across trials [41]. Yet, in the Roche et al. [40] study, the threshold was determined at the point when participants provided wrong responses on three trials with the same inter-onset-interval. Therefore, the lack of group differences reported by this study may be due to the perceptual threshold estimation method used. While these studies shine some light on auditory–perceptual abilities in children with DCD, more research is needed to determine whether these children indeed have auditory perceptual timing difficulties and whether this relates to duration timing, beat-based timing, or both.

### 3.2. Motor Timing

Eight studies examined motor timing in children with DCD, five of which employed a self-paced task, while three employed a continuation paradigm [44]. In a self-paced task, individuals select a tapping or other movement rate to maintain without any external sensory reference (e.g., auditory or visual cues). In contrast, a continuation paradigm requires participants to first synchronize their movements to a series of isochronous tones (synchronization phase) and then to continue their movements at the same frequency after the auditory cue stops (continuation phase). The level of motor consistency or variability during the continuation phase is thought to provide insight into internal timing mechanisms [45].

#### 3.2.1. Self-Paced Movements

As expected, children with DCD exhibited more variability in their motor timing across self-paced motor timing tasks, including simple tapping tasks [46] as well as bimanual [22,47] and interlimb movements [48,49]. When comparing hand–hand and hand–foot coordination patterns, children with DCD demonstrated greater variability in the latter during both the in-phase (limbs moving simultaneously) and anti-phase (limbs alternating) tasks [48]. Additionally, one study found that variability in self-paced motor timing increased among “clumsy” children when they were required to change their self-selected speed, particularly when they were instructed to slow down [46]. This is consistent with the literature showing that, in addition to exhibiting greater movement variability, 6–11-year-old children show a preference towards faster tapping tempos compared to adults [50,51], suggesting that there is a developmental progression from matching the faster frequencies to matching the slower ones even when mastering simple unpaced tapping tasks.

#### 3.2.2. Continuation Paradigm

All three studies that used the continuation paradigm found that “clumsy” children were more variable in maintaining a set frequency of tapping to a predefined rhythm in both unimanual [38,39] and bimanual rhythmic tapping tasks [52]. In one study, “clumsy” children were grouped based on the assumed etiology, and those with cerebellar soft signs showed increased difficulty in internal timing mechanisms compared to those with basal ganglia soft signs who were instead more variable on the force task [38]. Another study found that both the young “clumsy” children and young controls (6–7 years) were more variable than the older “clumsy” children and their age-matched controls (9–10 years) [39]. However, children in this study with motor difficulties always exhibited greater variability than their age-matched controls. Both studies discuss their results in the context of the information processing framework [53]. Specifically, they propose that the source of continuation motor timing difficulties may be a central timekeeping mechanism (i.e., internal clock). On the contrary, Geuze and Kalverboer [52] found no significant difference in the measures of tapping stability on a range of unimanual and bimanual tapping tasks between clinical groups. The reason for this may be their inclusion criteria, as they compared children who are “clumsy” with children with dyslexia and with age-matched children without reading or motor difficulties; however, all children were pooled from a school for children with specific learning difficulties. Therefore, it is possible that children across all groups had some level of motor challenges. 

#### 3.2.3. Intrinsic Feedback

Studies have also examined the influence of auditory and visual input on motor performance by manipulating the amount of task-intrinsic feedback, i.e., auditory and visual cues generated by the task itself. It is important to emphasize that these experiments can also be considered within the auditory–motor timing domain; however, since they did not specifically examine the effects of external sensory cues (e.g., coming from a metronome or a computer) on motor responses, we categorised them under the umbrella of motor timing. Those studies used noise-cancelling headphones and/or a blindfold to eliminate the participants’ ability to see or hear their movement actions [47,49]. While children with DCD exhibited increased variability compared to their age-matched controls and adult controls, the removal of self-generated sounds or vision during a self-paced tapping task [47] and a gross-motor coordination task [49] did not affect performance in any of the groups. Thus, these studies suggested there is no role of intrinsic visual and auditory perception in the consistency of motor timing. Of course, in these studies, it was not possible to eliminate tactile sensory feedback. Interestingly, children with DCD were substantially more variable when visual or visual–auditory intrinsic feedback was available, compared to auditory alone [47], suggesting that auditory–motor and visual–motor integration processes are distinct. Therefore, the availability of visual and/or auditory intrinsic feedback does not seem to affect the variability of motor coordination in self-paced tasks [47,49].

### 3.3. Auditory–Motor Timing

Auditory and motor brain networks interact during passive listening as well as during generation of motor movements to predictable rhythmic auditory stimuli, promoting integration of the auditory and motor systems [27,54]. Given that interactions between auditory and motor systems underlie many functional everyday behaviours, including speech, reacting to traffic noises, or note-taking during a lecture, vulnerabilities in auditory–motor timing may significantly affect a child’s adaptive functioning and quality of life. Auditory–motor coupling has commonly been studied using a simple finger-tapping paradigm. In this task, participants are asked to synchronize the finger taps of their dominant hand to an external auditory cue (e.g., metronome) [54,55,56,57]. The key indicator of accurate sensorimotor timing is tapping slightly ahead of the beat (a phenomenon called negative mean asynchrony) [55] which implies that, once the motor responses become entrained by the auditory stimuli, the participant anticipates rather than reacts to rhythmic auditory stimuli [58,59]. The primary finding of the studies that used synchronization paradigms was that children with DCD exhibited greater trial-level variability in the difference between their motor movement and the pacing stimuli, although the average synchronization accuracy (the ability to match tapping rate) across trials did not differ between groups [23,40,60].

#### 3.3.1. Synchronization Paradigm

Compared to their age-matched controls and adult controls, children with DCD had difficulties matching motor movements to auditory rhythms when producing bilateral antiphase movements [23] and when coordinating their limbs in a gross-motor coordination task [60]. They were most variable when matching the lowest frequency during a tapping task [23]. In contrast, during a gross-motor coordination task involving clapping and marching to an auditory beat, higher frequencies resulted in greater variability [60]. These differences may be due to different biomechanical constraints that underlie discrete versus continuous rhythmic movements. Additionally, developmental effects between the groups were observed in both studies [23,60], manifested by greater synchronization accuracy and negative mean asynchrony (tapping ahead of the beat) in adult controls, while children with and without DCD tended to tap behind the beat. Across studies, when the frequency of the auditory cue was sped up, children with DCD showed less stable motor patterns in unimanual [46] and bimanual in-phase and anti-phase tapping tasks [22] as well as when performing hand–hand and hand–foot coordination patterns, with the least stability in the latter [48]. The differential effect of frequency rate is further explored in the discussion.

Roche et al. [40] examined auditory–motor synchronization abilities using a task that required children to adapt their responses to perceptible and subliminal rhythmic changes in auditory stimuli. They found that children in both groups were equally able to adjust their tapping to gradual and abrupt changes in phasing, although children with DCD had greater trial-by-trial variability in both conditions. In other studies that aimed to differentiate subconscious and conscious adaptations in healthy adults, it was reported that frequency fluctuations of <5% (and possibly 7%) are perceived on a subliminal level and, thus, result in subconscious adaptations of tapping patterns, whereas large frequency fluctuations of 20% are reliably detected and prompt conscious adaptations of motor actions [61,62,63]. For that reason, it is possible that the stimulus phase change of 12.5% in Roche et al. [40] might have elicited preconscious rather than conscious adaptations for some participants. Finally, a synchronization task was employed to examine the ability to selectively inhibit symmetrical movements (i.e., mirror movements) while switching from bimanual to unimanual tapping. In this experiment [64], both control and DCD groups showed improvements in their ability to inhibit left finger taps with practice; however, children with DCD produced a significantly higher number of supplementary movements and showed no age-related improvement in their capacity to inhibit left finger taps. 

#### 3.3.2. Reaction Time

Reaction time (RT) tasks have also been used as an indicator of auditory–motor processes [65,66,67]. For example, RT was measured to assess how auditory stimuli influence the performance in an aiming task (i.e., moving a finger from the start position to the target button) as part of a multisensory paradigm [67]. Reaction times to auditory stimuli were faster compared to visual but slower compared to multisensory auditory–visual stimuli in both groups, though children with DCD reacted slower in all conditions. O’Brien et al. [65] also found that children with DCD provided slower responses than controls under all three sensory conditions (i.e., auditory, visual, and vibrotactile); however, their performance did not result in more performance errors and was not more variable compared to controls. The authors noted that all children received extensive practice prior to the experiment, which might have contributed to the lack of group difference. Interestingly, young children with DCD (6–7 years) exhibited faster responses to auditory stimuli than did their age-matched controls, and also had significantly higher receptive vocabulary scores. This effect was not found in older groups (9–10 years). Additionally, Rosenblum and Regev [66] found that children with DCD exhibited significantly longer response times when adjusting their hands and feet to the auditory beat, which also predicted their handwriting performance.

#### 3.3.3. Sensorimotor Learning

Lastly, a recent study by Blais et al. [21] examined the effect of auditory stimuli on learning, retention, and reactivation of perceptual–motor non-isochronous sequences. Children were given 30 practice trials in which they synchronized their movements to patterns of auditory or visual stimuli followed by an immediate and delayed recollection of the patterns without external cues. While both groups of children showed more significant improvements in temporal accuracy and stability during practice with auditory compared to visual stimuli, children with DCD demonstrated a significant increase in errors during recall of auditory versus visual patterns (i.e., after the auditory stimuli were removed). In other words, while learning and retention of a visual sequence were preserved, auditory sequence retention appears to be a specific vulnerability in children with DCD. Therefore, it is plausible that children with DCD benefit more from auditory information during learning but not when memorizing a temporal sequence.

**Table 2:** Key characteristics of included studies.

Author(s)	Sample (*N*) (Female/Male)	Age (Years)	Motor Test (Cut-Off)	Experimental Task	Timing Modality	Key Findings
Geuze & Kalverboer, 1987 [46]	“Clumsy”: 17 (4 F, 13 M) Controls: 17 (5 F, 12 M)	7–11	Teacher’s ratings on Motor Impairment Test	Self-paced Synchronization/adaptation: unimanual tapping (64, 84, 104 bpm)	Motor, Auditory- motor	Self-paced: ↑ variability (especially slow) Synchronization: ↑ variability (especially fast) Similar variability on both tapping tasks
Lundy-Ekman et al., 1991 [38]	“Clumsy”: 25 (11 BG signs, 14 CB signs) Controls: 14	7–8	Screening for soft and hard signs; BOT–Short Form	Perception: duration timing Continuation: unimanual tapping Force	Auditory, Motor	(CB) Perception: ↓ accuracy (CB) Continuation: ↑ variability (BG) Force: ↑ variability
Williams et al., 1992 [39]	“Clumsy”: 12 (6 Y, 6 O) Controls: 13 (6 Y, 7 O)	Y: 6–7 O: 9–10	BOT–Short Form	Perception: duration and loudness Continuation: unimanual tapping Force	Auditory, Motor	Perception: ↓ accuracy Continuation: ↑ variability Age differences in variability
Geuze & Kalverboer, 1994 [52]	“Clumsy”: 11 Dyslexic: 12 “Controls”: 12	7–12	TOMI: DCD > 4.5, Control < 4	Continuation: unimanual, alternating bimanual, 2:1 or 1:2 asymmetrical tapping	Motor	Continuation: ↑ variability (especially slow) No significant difference: “Clumsy” → Dyslexic → “Controls” All groups attended a school for children with specific learning difficulties
Volman & Geuze, 1998 [22]	DCD: 24 Controls: 24	7–12	MABC < 15th percentile Touwen Test	Self-paced bimanual tapping: in-phase/anti-phase, perturbation Synchronization/adaptation: anti-phase hand–hand patterns	Motor, Auditory- motor	Self-paced: ↑ variability of coordination pattern Synchronization/adaptation: ↑ variability The critical frequency ↓ in DCD than controls
Volman et al., 2006 [48]	DCD: 10 Controls: 16	5–8	MABC < 15th percentile	Self-paced: in-phase, anti-phase Synchronization/adaptation (anti-phase): hand–hand, hand–foot (ipsilateral and contralateral)	Motor, Auditory- motor	Self-paced: ↑ variability Scaled frequency: ↑ variability (all combinations) Non-homologous patterns less stable
Whitall et al., 2006 [60]	DCD: 10 Controls: 8 Adult controls: 10	6–8 18–30	MABC < 10th percentile	Synchronization: gross-motor coordination (clapping, marching) at 0.8, 1.2, 1.6 and 2.0 Hz	Auditory- motor	Synchronization: ↑ variability (especially fast) Similar mean error: DCD → Controls → Adults All groups: hands more stably locked to the beat
Whitall et al., 2008 [23]	DCD: 10 Controls: 10 Adult controls: 10 (3 F, 7 M)	6–8 21–35	MABC < 15th percentile	Synchronization: bilateral antiphase finger tapping at 0.8, 1.6, 2.4, 3.2 Hz per finger	Auditory- motor	Synchronization: ↑ variability (especially slow) All groups able to modulate their finger frequency Adults tapping ahead→ DCD/controls behind
Mackenzie et al., 2008 [49]	DCD: 11 Controls: 7 Adult controls: 10	6–8 18–30	MABC < 10th percentile	Self-paced: gross-motor coordination (clapping in-phase to marching) with vision and hearing, no vision, no hearing, and without both	Motor	↑ variability but no significant condition effects All groups: ↓ variability marching vs. clapping DCD/controls: foot–foot coherence equally stable
O’Brien et al., 2008 [65]	Y DCD: 7 Y controls: 10 O DCD: 9 O controls: 10	Y: 6–7 O: 9–10	MABC < 15th percentile	Synchronization RT to visual, auditory, and vibrotactile stimuli: compatible/incompatible	Auditory- motor	DCD RTs ↑ under all three sensory conditions No difference in RT variability or errors Young DCD: Faster to auditory than controls
Roche et al., 2011 [47]	DCD: 10 Controls: 10 Adults: 10 (3 F, 7 M)	6–8 21–35	MABC < 15th percentile PANESS	Self-paced anti-phase tapping: vision and audition (VA), no vision (A), no audition (V), no vision and audition	Motor	DCD ↓ accuracy, ↑ variability vs. controls/adults Sensory conditions did not affect performance DCD ↑ variability with V or VA feedback
Rosenblum & Regev, 2013 [66]	DCD: 21 Controls: 21 (8 F, 13 M)	7–10	MABC < 15th percentile	Synchronization to IM: 14 tasks Handwriting: three tasks	Auditory- motor	RTs ↑ for 11 out of 14 IM tasks Handwriting performance variance ↑ Mean total RT predicts the handwriting variance
Tallet et al., 2013 [64]	DCD:12 (4 F, 8 M) Controls: 12 (4 F, 8 M)	7–10	MABC < 15th percentile	Synchronization/Inhibition: switching from bimanual symmetrical tapping to unimanual tapping	Auditory- motor	Similar mean self-selected tempo DCD number of supplementary taps significantly ↑ Left finger inhibition: improvement through trials
Coats et al., 2015 [67]	DCD: 10 (4 F, 6 M) Controls: 10 (5 F, 5 M)	7–10	MABC < 9th percentile	Synchronization RT (aiming task): unimodal visual (V), auditory (A), and bimodal condition (AV)	Auditory- motor	V (slowest) → A→ AV stimulus (fastest) Movement planning: both groups benefit (AV) Movement control: only controls benefit (AV)
Roche et al., 2016 [40]	DCD: 24 (19) Controls: 22 (17)	6–11	MABC < 5th percentile PANESS	Perception: beat-based timing Synchronization/adaptation (bimanual anti-phase tapping): gradual/abrupt conditions	Auditory, Auditory- motor	Auditory perception: No difference Synchronization/adaptation: No difference DCD significantly more variable in all conditions
Blais et al., 2021 [21]	DCD: 12 Controls: 20	8–12	MABC < 5th percentile	Synchronization: learning, retention, and reactivation (auditory or visual) Control task: Isochronous sequence	Auditory- motor	Practice: ↑ stability audio-motor sequence Immediate retention: DCD ↑ errors auditory Visuo-motor sequence equally learned, retained and reactivated in both groups

Note. DCD = developmental coordination disorder; Clumsy = the term used to describe children with motor coordination difficulties before “DCD” started being used in 1994; BG = Basal ganglia; CB = cerebellum; Y = young; O = old; TOMI = Test of Motor Impairment; BOT–Short Form = Bruininks–Oseretsky Test of Motor Proficiency; MABC = The Movement Assessment Battery for Children [68]; MABC percentiles = the cut-off point of < 16th percentile indicates motor coordination difficulties (lower percentile indicates more severe difficulties); PANESS = Physical and Neurological Examination of Subtle Signs [69]; F = female; M = male; RT = reaction time; A = auditory; V = visual; AV = auditory–visual condition; IM = Interactive Metronome [70].

## 4. Discussion

To our knowledge, this is the first review of associations between auditory–perceptual abilities and motor timing in DCD. We identified 16 studies that were included based on the timing modality studied (i.e., auditory–perceptual, motor, or auditory–motor). Only three studies assessed auditory perceptual abilities of children with DCD separate from their motor or auditory–motor performance, leading to insufficient and inconsistent findings. Within the motor timing domain, five studies employed a self-paced task and three used a continuation paradigm, while eleven studies employed synchronization paradigms or reaction time tasks as an indicator of auditory–motor processes. The key finding of these studies is that children with DCD exhibit more variability both in maintaining a set frequency of tapping to a predefined rhythm as well as when matching their movement to an external auditory cue, compared to their age-matched controls.

### 4.1. Motor Variability with and without External Auditory Stimuli

This review further confirms that variability in and slowness of motor response are key characteristics of DCD, regardless of the experimental task. From a theoretical standpoint, perceptual–motor timing abilities have been examined using two dominant perspectives: information processing and the dynamical systems theory. The information processing framework has mainly been considered in earlier studies using the Wing–Kristofferson timekeeper model [44]. Those studies suggest that the increased motor variability observed in children with DCD is a result of a deficit in motor programming (i.e., central timekeeping processes) rather than processes related to motor execution [38,39,46,52]. In contrast, the dynamic pattern perspective explains those coordination difficulties in terms of the interplay between the internal and external forces [71,72,73] where reduced temporal motor stability indicates difficulties in dynamic movement control [22,23,40,48]. Given that these two theoretical frameworks account for different aspects of perceptual-motor timing, their relevance primarily depends on the task demands [74].

Taken together, our results are consistent in finding that children with DCD have difficulties coordinating their movements both with external auditory stimuli [23,40,60] and when maintaining a movement pattern without external cues [38,39,52]. The overall slowness of movements has been most evident in studies that used reaction-time paradigms [65,66,67]. It is interesting to note that there were no significant differences between the groups with respect to the mean self-selected tapping frequency [22,47,64]. Combined, the extant literature also points to a differential effect of frequency rate on motor variability across tasks. For example, children with DCD seem to become more variable (i.e., less stable) at faster frequencies when matching the auditory beat in tapping synchronization paradigms [22,46]. In contrast, slower frequencies present more difficulty during unpaced tapping tasks [46,52]. Although these studies often make inferences about the auditory perceptual abilities of children with DCD based on either a motor or an auditory–motor task, our findings indicate a paucity of research focusing specifically on the role of auditory perception in motor coordination tasks. 

As noted earlier, auditory–motor variability could arise as a consequence of inefficient time perception, motor execution, and/or sensorimotor coupling. Therefore, in addition to testing auditory timing separately, future studies should compare motor variability between paced and unpaced tasks to distinguish whether external auditory cues contribute to a more or less stable performance. Since auditory processing develops earlier [75] and is associated with faster reaction times compared to visual information [76], auditory interventions may be strong candidates for improving motor coordination in children with DCD. This knowledge could be clinically relevant considering the implications of rhythmic auditory stimuli seen in movement rehabilitation with people with Parkinson’s disease [77,78], traumatic brain injury [79], stroke [80,81], and cerebral palsy [82]. These studies suggest that rhythmic auditory cues increase temporal stability and speed, facilitating the planning and execution of movements in persons with movement disorders, thus proving to be an effective therapeutic tool.

### 4.2. Auditory Perceptual Abilities in Children with DCD

Only three out of sixteen studies tested perceptual discrimination abilities using an auditory timing task in addition to motor-related paradigms, and their results are inconclusive. Two of those studies found that children with DCD have decreased acuity (i.e., higher thresholds) on duration timing tasks, while the study testing perceptual thresholds for beat-based timing found no group difference. These opposing results are likely due to the differences in the psychophysical procedures and inclusion criteria used. We identified two additional studies that examined auditory perceptual abilities using electrophysiology measures in children aged 6–7 years with (probable) DCD but did not meet the inclusion criteria for this review. Holeckova et al. [83] found differences in event-related potentials [84] during a passive auditory oddball task indicating that children with DCD have reduced ability to detect and evaluate deviations in the stimuli (they reported no mismatch negativity response and a reduction of P3 amplitude in this group). Chang et al. [85] used a more comprehensive design and measured perceptual discrimination thresholds for duration timing, rhythm timing, and pitch (control) using both behavioural and electrophysiology measures. They found that children at risk for DCD have larger discrimination thresholds (i.e., decreased acuity) for both the duration and rhythm-based timing compared to controls, which was also reflected by the delayed mismatch negativity for duration deviations and delayed P3a latencies in response to rhythm deviants. Both studies provide support for the auditory–perceptual timing hypothesis [24], which suggests that, in addition to motor deficits, auditory–perceptual timing deficits appear to be the core of the disorder as well; however, current evidence is insufficient, and more research is needed to understand the brain-behaviour association related to auditory–perceptual abilities and whether potential difficulties apply to both duration- and beat-based timing.

### 4.3. Limitations and Future Directions

To capture all relevant studies, the date of publication was not an exclusion criterion in our review. However, we recognize that the criteria for study sample inclusion differed in research conducted before 1994, when an international panel of experts agreed to the term “developmental coordination disorder” to describe children with significant motor coordination difficulties. We encountered a wide variation in terminology across earlier studies, and kept the original terms (i.e., clumsy) when describing their findings. Since then, a score < 16th percentile on The Movement Assessment Battery for Children (MABC) [68] has been used as a cut-off point for diagnosing DCD (lower percentile indicates greater severity), in addition to other criteria outlined in The Diagnostic and Statistical Manual of Mental Disorders (5th ed.) [1]. As shown in Table 2, even among studies that used the MABC, the diagnostic cut-off scores varied from the 5^th^ to the 15th percentiles. Given the variation in the terminology and diagnostic criteria used, direct comparisons among studies should be interpreted with caution. Moreover, only a few studies screened for possible comorbidities; therefore, it is possible that performance differences may have been confounded with difficulties in other domains such as attention or working memory. Only four studies controlled for the effects of prior music training on the auditory–motor performance, and this was done mainly for adult controls [21,23,47,49]. The degree to which music training can induce changes in auditory–motor coupling in children with DCD has yet to be studied. Nevertheless, research suggests that extensive music training can gradually affect the manner in which the periodicities of a musical rhythm are perceived and performed, thereby enhancing auditory–motor coupling [25,86]. Therefore, future studies should assess and control for possible effects of music training. Finally, considering that many of the studies provided limited information regarding individual demographic and environmental differences, we currently do not know the extent to which the results are generalizable to the overall population.

In conclusion, the selected studies provide important insights regarding the contribution of three timing modalities that underlie efficient auditory–motor coupling. However, caution is needed when comparing and interpreting these results due to the considerable methodological heterogeneity that is present in these studies (see Table 2), especially given that children with DCD are a rather heterogeneous group. Finally, all the identified studies employed behavioural measures only. Therefore, insights regarding the brain-behaviour correlates of auditory–motor coupling are limited and necessitate future research.

## 5. Conclusions

This review aimed to summarize what is known about auditory–motor timing in children with DCD. The extent to which auditory perception relates to motor variability in children with DCD has largely been understudied. Therefore, limited conclusions can be made about the specific contributions of auditory perceptual abilities in this population, especially due to the heterogeneity of DCD as well as the methodological heterogeneity of the included studies. Further research is needed to evaluate the associations between paced and unpaced tasks so that the distinct contribution of auditory timing can be discerned. Considering that auditory interventions show promise for improving movement in other clinical samples [30], understanding the contribution of auditory perception and the effects of auditory stimuli on motor control in children with DCD will help inform whether auditory–perceptual training might be a plausible therapeutic intervention.

## Figures and Tables

**Table 1:** Search strategy used for selection of scientific articles in MEDLINE.

**Database**	**MEDLINE** (Ovid)				
**Coverage**	Medicine and Health				
**Limits**	Publication Type: “Article”, Language: “English”, Age: “Children <18”				
**Search Query**	**Population: DCD**		**AND**	**Concept: Auditory-Motor Timing**	
	**Headings**	**Keywords**		**Headings**	**Keywords**
	exp Motor Skills Disorders/	Developmental coordination disorder.mp. DCD.mp. (developmental coordination adj3 (disorder* or problem* or dysfunction* or difficult* or impairment* or deficit*)).mp. Motor skill* disorder.mp. (clums* adj3 child).mp. ((movement* or motor) adj1 (difficult* or deficit*)).mp. Dyspraxia.mp.		exp Acoustic Stimulation/	(audi* adj3 motor*).mp. auditory motor adj3 (synchron* or coupling).mp. ((auditory or audio or percept* or rhythm*) adj7 (motor or rhythm* or cue* or tap* or stimul* or action or synchron* or entrainment or coupling or adaptation* or interaction* or integration or performance or timing or processing)).mp. tap* adj5 (audi* or rhythm* or finger).mp.
**Results**	371				

## Data Availability

Not applicable.

## Supplementary Materials

The following supporting information can be downloaded at: https://www.mdpi.com/article/10.3390/brainsci13050729/s1, Table S1: Search Strategy; Table S2: Title and Abstract Screening; and Table S3: Full-Text Screening [83,85,87,88,89,90,91,92,93,94,95,96,97,98,99,100].
