# Supplementary material for: Auditory–Perceptual and Auditory–Motor Timing Abilities in Children with Developmental Coordination Disorder: A Scoping Review
Source: Brain Sci. 2023 Apr 27;13(5):729. doi: 10.3390/brainsci13050729 (PMC10216155; doi:10.3390/brainsci13050729)
Supplement: Supplementary file 1 [file brainsci-13-00729-s001.zip › brainsci-2337006-supplementary.pdf]

Supplementary Table S1. Search Strategy.

<table>
<tr><td>Database</td><td colspan="5">Medline (Ovid)</td></tr>
<tr><td>Coverage</td><td colspan="5">Medicine and Health</td></tr>
<tr><td>Date</td><td colspan="5">April 20, 2022</td></tr>
<tr><td>Limits</td><td colspan="5">Publication Type: “Article”, Language: “English”, Age: “Children <18”</td></tr>
<tr><td rowspan="3">Search Query</td><td colspan="2">Population: Developmental Coordination Disorder
(combined with ‘OR’)</td><td rowspan="3">AND</td><td colspan="2">Concept: Auditory-Motor Timing
(combined with ‘OR’)</td></tr>
<tr><td>Subject Headings</td><td>Keywords</td><td>Subject Headings</td><td>Keywords</td></tr>
<tr><td>exp Motor Skills Disorders/</td><td>Developmental coordination disorder.mp.
DCD.mp.
(developmental coordination adj3 (disorder* or problem* or dysfunction* or difficult* or impairment* or deficit*)).mp.
Motor skill* disorder.mp.
(clums* adj3 child).mp.
((movement* or motor) adj1 (difficult* or deficit*)).mp.
Dyspraxia.mp.</td><td>exp Acoustic Stimulation/</td><td>(audi* adj3 motor*).mp.
auditory motor adj3 (synchron* or coupling).mp.
((auditory or audio or percept* or rhythm*) adj7 (motor or rhythm* or cue* or tap* or stimul* or action or synchron* or entrainment or coupling or adaptation* or interaction* or integration or performance or timing or processing)).mp.
tap* adj5 (audi* or rhythm* or finger).mp.</td></tr>
<tr><td>Number of Hits</td><td colspan="5">371</td></tr>
</table>

<table>
<tr><td>Database</td><td colspan="5">Embase (Ovid)</td></tr>
<tr><td>Coverage</td><td colspan="5">Medicine and Health</td></tr>
<tr><td>Date</td><td colspan="5">April 20, 2022</td></tr>
<tr><td>Limits</td><td colspan="5">Publication Type: “Article”, Language: “English”, Age: “Children <18”</td></tr>
<tr><td rowspan="3">Search Query</td><td colspan="2">Population: Developmental Coordination Disorder
(combined with ’OR’)</td><td rowspan="3">AND</td><td colspan="2">Concept: Auditory-Motor Timing
(combined with ‘OR’)</td></tr>
<tr><td>Subject Headings</td><td>Keywords</td><td>Subject Headings</td><td>Keywords</td></tr>
<tr><td>exp developmental coordination disorder/</td><td>Motor skill* disorder.mp.
DCD.mp.
(developmental coordination adj3 (disorder* or problem* or dysfunction* or difficult* or impairment* or deficit*)).mp.
dyspraxia.mp.
(clums* adj3 child).mp.
((movement* or motor) adj1 (difficult* or deficit*)).mp.</td><td>exp auditory stimulation/</td><td>(audi* adj3 motor*).mp.
auditory motor adj3 (synchron* or coupling).mp.
((auditory or audio or percept* or rhythm*) adj7 (motor or rhythm* or cue* or tap* or stimul* or action or synchron* or entrainment or coupling or adaptation* or interaction* or integration or performance or timing or processing)).mp.
tap* adj5 (audi* or rhythm* or finger).mp.</td></tr>
<tr><td>Number of Hits</td><td colspan="5">361</td></tr>
</table>

**Database**	**PsycInfo** (Ovid)
**Coverage**	Psychology

**Date**	April 20, 2022				
**Limits**	Publication Type: “Article”, Language: “English”, Age: “Children <18”				
**Search Query**	Population: Developmental Coordination Disorder<br>(combined with ‘**OR**’)		**AND**	Concept: Auditory-Motor Timing<br>(combined with **‘OR’**)	
	Subject Headings	***Keywords***		***Subject Headings***	***Keywords***
	exp Dyspraxia/	Motor skill* disorder.mp.<br>DCD.mp.<br>(developmental coordination adj3 (disorder* or problem* or dysfunction* or difficult* or impairment* or deficit*)).mp.<br>(clums* adj3 child).mp.<br>((movement* or motor) adj1 (difficult* or deficit*)).mp.		exp Auditory Stimulation/	(audi* adj3 motor*).mp.<br>auditory motor adj3 (synchron* or coupling).mp.<br>((auditory or audio or percept* or rhythm*) adj7 (motor or rhythm* or cue* or tap* or stimul* or action or synchron* or entrainment or coupling or adaptation* or interaction* or integration or performance or timing or processing)).mp.<br>tap* adj5 (audi* or rhythm* or finger).mp.
**Number of Hits**	728				

**Database**	**CINAHL** (EBSCO)				
**Coverage**	Nursing & allied health sciences				
**Date**	April 20, 2022				
**Limits**	Publication Type: “Article”, Language: “English”, Age: “Children <18”				
**Search Query**	Population: Developmental Coordination Disorder<br>(combined with ‘**OR**’)		**AND**	Concept: Auditory-Motor Timing<br>(combined with **‘OR’**)	
	Subject Headings	***Keywords***		***Subject Headings***	***Keywords***
	MH Motor Skills Disorders	developmental coordination disorder OR dyspraxia OR DCD or coordination disorder OR developmental coordination OR clumsiness OR clumsy child OR movement difficult* OR movement deficit* OR motor difficult* OR motor deficit*		MH Auditory Perception	auditory OR audio OR percept* action OR sensorimotor OR auditory motor<br>motor OR synchron* OR coupling OR rhythmic OR cue* OR tap* OR entrainment OR adaptation* OR interaction* OR integration OR stimul* OR performance OR timing OR processing
**Number of Hits**	108				

**Database**	**Scopus**			
**Coverage**	Interdisciplinary			
**Date**	April 20, 2022			
**Limits**	Publication Type: “Article”, Language: “English”, Age: “Children <18”			
	Population: Developmental Coordination Disorder<br>(combined with ‘**OR**’)	**AND**	Concept: Auditory-Motor Timing<br>(combined with **‘OR’**)	
	Keywords		***Keywords***	

Search Query	{developmental coordination disorder} OR {DCD} OR {dyspraxia} OR "motor skill* disorder" OR "clumsiness" OR "coordination disorder" OR "developmental coordination" OR "clumsy child" OR "movement difficult*" OR "movement deficit*" OR "motor difficult*" OR "motor deficit*"		"auditory motor synchron*" OR "audi* motor" OR "percept* action" OR "auditory stimul*" OR "rhythmic synchron*" OR "sensorimotor synchron*" OR "rhythm* tap*" OR "auditory cue*" OR "auditory motor coupling" OR "auditory motor entrainment" OR "audi* motor adaptation*" OR "audi* motor interaction*" OR "audi* motor integration" OR "audi* motor timing" OR "audi* motor processing" OR "audi* motor performance"
Number of Hits	105		

Supplementary Table S2: Title and Abstract Screening.

	**Exclusion Criteria**	**YES**	**NO**	**Maybe**
1	Does the title/abstract identify the paper as a review or a survey study?			
2	Does the title/abstract identify the study as a conference proceeding, meeting minute, commentary, postscript, letter or in video format?			
3	Are participants in the study adults (above 18 y.o.)?			
4	Is the paper published in a language other than English?			
	**If "Yes" to any of the above, exclude the study.**			
	**Inclusion Criteria**			
5	Does the title/abstract mention the effects of auditory stimuli on motor performance?			
6	Does the title/abstract mention children (<18 years) with motor coordination difficulties?			

If the reviewer's answer is "No" or "Maybe" to questions 1-4, a full-text article will be reviewed for decision-making on this level. If the reviewer's answer is "Yes" or "Maybe" to questions 5 and 6, the full-text article will be reviewed for decision-making on this level.

Supplementary Table S3. Full-Text Screening.

	**Reasons for Exclusion**	✓
1	The study did not report the effects of auditory stimuli on motor performance.	
2	The primary outcome measure was not related to motor performance (e.g., the primary goal of the study was an evaluation of a treatment method).	

	**Sources Excluded**	**Primary Reason of Exclusion**
1	Biotteau, M., Chaix, Y., & Albaret, J. M. (2015). Procedural learning and automatization process in children with developmental coordination disorder and/or developmental dyslexia. *Human movement science*, *43*, 78-89.	1
2	Caçola, P., Getchell, N., Srinivasan, D., Alexandrakis, G., & Liu, H. (2018). Cortical activity in fine-motor tasks in children with developmental coordination disorder: a preliminary fNIRS study. *International Journal of Developmental Neuroscience*, *65*, 83-90.	1
3	Chang, A., Li, Y. C., Chan, J. F., Dotov, D. G., Cairney, J., & Trainor, L. J. (2021). Inferior auditory time perception in children with motor difficulties. *Child Development*, *92*(5), e907-e923.	2

4	Cosper, S. M., Lee, G. P., Peters, S. B., & Bishop, E. (2009). Interactive Metronome training in children with attention deficit and developmental coordination disorders. *International Journal of Rehabilitation Research*, *32*(4), 331-336.	2
5	de Castelnau, P., Albaret, J. M., Chaix, Y., & Zanone, P. G. (2008). A study of EEG coherence in DCD children during motor synchronization task. *Human Movement Science*, *27*(2), 230-241.	1
6	Hession, C. E., Law Smith, M. J., Watterson, D., Oxley, N., & Murphy, B. A. (2019). The Impact of equine therapy and an audio-visual approach emphasizing rhythm and beat perception in children with developmental coordination disorder. *The Journal of Alternative and Complementary Medicine*, *25*(5), 535-541.	2
7	Holeckova, I., Cepicka, L., Mautner, P., Stepanek, D., & Moucek, R. (2014). Auditory ERPs in children with developmental coordination disorder. *Activitas Nervosa Superior*, *56*(1), 37-44.	2
8	King, B. R., Kagerer, F. A., Harring, J. R., Contreras-Vidal, J. L., & Clark, J. E. (2011). Multisensory adaptation of spatial-to-motor transformations in children with developmental coordination disorder. *Experimental brain research*, *212*(2), 257-265.	1
9	Lê, M., Blais, M., Jucla, M., Chauveau, N., Maziero, S., Biotteau, M., ... & Tallet, J. (2021). Procedural learning and retention of audio-verbal temporal sequence is altered in children with developmental coordination disorder but cortical thickness matters. *Developmental Science*, *24*(1), e13009.	1
10	Leemrijse, C., Meijer, O. G., Vermeer, A., Adèr, H. J., & Diemel, S. (2000). The efficacy of Le Bon Départ and Sensory Integration treatment for children with developmental coordination disorder: a randomized study with six single cases. *Clinical Rehabilitation*, *14*(3), 247-259.	2
11	Mikami, M., Hirota, T., Takahashi, M., Adachi, M., Saito, M., Koeda, S., ... & Yamada, J. (2021). Atypical sensory processing profiles and their associations with motor problems in preschoolers with developmental coordination disorder. *Child Psychiatry & Human Development*, *52*(2), 311-320.	1
12	Niklasson, M., Niklasson, I., & Norlander, T. (2009). Sensorimotor therapy: using stereotypic movements and vestibular stimulation to increase sensorimotor proficiency of children with attentional and motor difficulties. *Perceptual and Motor Skills*, *108*(3), 643-669.	1
13	Niklasson, M., Norlander, T., Niklasson, I., & Rasmussen, P. (2017). Catching-up: Children with developmental coordination disorder compared to healthy children before and after sensorimotor therapy. *PloS one*, *12*(10), e0186126.	1
14	Piek, J. P., & Skinner, R. A. (1999). Timing and force control during a sequential tapping task in children with and without motor coordination problems. *Journal of the International Neuropsychological Society*, *5*(4), 320-329.	1
15	Sartori, R. F., Valentini, N. C., Nobre, G. C., & Fonseca, R. P. (2021). Motor and verbal inhibitory control: Development and validity of the go/No-Go app test for children with development coordination disorder. *Applied Neuropsychology: Child*, *10*(4), 359-368.	1
16	Volman, M. J. M., & Geuze, R. H. (1998). Stability of rhythmic finger movements in children with a developmental coordination disorder. *Motor Control*, *2*(1), 34-60.	1
